# Genome-wide Identification, Characterization, and Expression Analysis of PHT1 Phosphate Transporters in Wheat

**DOI:** 10.3389/fpls.2017.00543

**Published:** 2017-04-11

**Authors:** Wan Teng, Yan-Yan Zhao, Xue-Qiang Zhao, Xue He, Wen-Ying Ma, Yan Deng, Xin-Ping Chen, Yi-Ping Tong

**Affiliations:** ^1^The State Key Laboratory for Plant Cell and Chromosome Engineering, Institute of Genetics and Developmental Biology, Chinese Academy of SciencesBeijing, China; ^2^Institute of Tropical Agriculture and Forestry, Hainan UniversityHaikou, China; ^3^Research Center of Resource, Environment and Food Security, China Agricultural UniversityBeijing, China

**Keywords:** wheat (*Triticum aestivum*), *PHT1* genes, genome-wide analysis, phosphate transporter, phosphorus uptake, phosphate-starvation response

## Abstract

The PHT1 family of phosphate (Pi) transporters mediates phosphorus (P) uptake and re-mobilization in plants. A genome-wide sequence analysis of *PHT1* genes in wheat (*Triticum aestivum*) was conducted, and their expression locations and responses to P availability were further investigated. We cloned 21 *TaPHT1* genes from the homologous alleles at *TaPHT1.1* to *1.10* through screening a BAC library and amplifying genomic sequences. The TaPHT1 transporters were clustered into five branches in the phylogenetic tree of PHT1 proteins, and the *TaPHT1* genes from a given branch shared high similarities in sequences, expression locations, and responses to P availability. The seven tested *PHT1* genes all showed Pi-transport activity in yeast (*Saccharomyces cerevisiae*) cells grown under both low Pi and high Pi conditions. The expression of *TaPHT1*.*1*/*1.9, 1.2*, and *1.10* were root specific. The expression of these *TaPHT1* genes at flowering positively correlated with P uptake after stem elongation across three P application rates and two wheat varieties in a field experiment. Therefore, modification of *PHT1* expression may improve P use efficiency in a broad regime of P availability.

## Introduction

Phosphorus (P) is one of the essential macronutrients for plant growth and development, and it takes part in cellular macromolecules, energy transfer reactions, and cellular metabolism. Efficient acquisition of phosphate (Pi) from soil combined with efficiency translocation of Pi within plants is essential for plants to maintain adequate levels of cellular Pi necessary for normal function (Raghothama and Karthikeyan, 2005). Although total P in soils is abundant, the soluble phosphate (Pi) is often low (Bieleski, 1973; Rausch and Bucher, 2002), and therefore plants often encounter a scarcity of Pi in soils of both agricultural and natural systems (Raghothama, 1999, 2000). As there is a large difference between Pi levels in plant cells (mM) and soil solution (μM), plants need to acquire Pi against a steep concentration gradient across the plasma membrane (Smith et al., 2003; Raghothama and Karthikeyan, 2005). The transmembrane transport of Pi from soils into plant cells requires a high-affinity, energy-driven transport mechanism (Smith et al., 2003). The PHT1 family of plant Pi transporters is assumed to play the predominant roles in this transmembrane transport process. These proteins are characterized by 12 membrane-spanning domains which are similar to PHO84, a high-affinity Pi transporter from yeast (*Saccharomyces cerevisiae*) (Muchhal et al., 1996; Rausch and Bucher, 2002).

There are four PHOSPHATE TRANSPORTER (PHT) families in plants: PHT1, PHT2, PHT3, and PHT4 which are located on plasma membrane, plastid inner membrane, mitochondrial inner membrane, and Golgi-compartment, respectively (Lopez-Arredondo et al., 2014). Under the stress of P-starvation, the expression of *PHT1* genes are strongly induced to increase the ability of the roots in acquiring P from soils and remobilize P within plants (Smith et al., 2003; Raghothama and Karthikeyan, 2005). A large number of *PHT1* transporters have been identified in many plant species and show differences in expression locations and affinities for Pi (Nussaume et al., 2011). Nine *PHT1* genes in Arabidopsis (*Arabidopsis thaliana*) have been identified. *AtPHT1.1* and *AtPHT1.4* are highly expressed at the root–soil interface, including the epidermis, root hair cells, and the root cap under low P conditions (Mudge et al., 2002), and they are the major genes responsible for Pi acquisition by roots in both high and low P supplies (Misson et al., 2004; Shin et al., 2004; Catarecha et al., 2007). *AtPHT1.8* and *AtPHT1.9* are likely to act sequentially in the interior of the plant during the root-to-shoot translocation of Pi and are involved in root-to-shoot translocation of Pi (Lapis-Gaza et al., 2014). There are 13 *PHT1* members in the rice (*Oryza sativa*) genome, and some of them have been functionally characterized, including *OsPHT1.1* (Sun et al., 2012), *OsPHT1.2* and *OsPHT1.6* (Ai et al., 2009), *OsPHT1.4* (Ye et al., 2015), and *OsPHT1.8* (Jia et al., 2011; Li et al., 2015). For example, *OsPHT1.6* is expressed in both epidermal and cortical cells of the younger primary and lateral roots and encodes as a high-affinity transporter with a broad role in Pi uptake and translocation throughout the plant, whereas *OsPHT1.2* is localized exclusively in the stele of primary and lateral roots and functions as a low-affinity transporter responsible for Pi translocation (Ai et al., 2009). Barley (*Hordeum vulgare*) is a close relative to wheat (*Triticum aestivum*). To date, 11 *PHT1* genes have been reported in barley. The *HvPHT1.1* and *HvPHT1.2* promoters drive the expression of β-*glucuronidase* (*GUS*) and *green fluorescent protein* (*GFP*) reporter genes in epidermal and cortex cells as well as vascular tissues of roots (Schunmann et al., 2004). When expressed in *Xenopus laevis* oocytes, HvPHT1.1 is confirmed to be a high-affinity transporter with a very low *K*_m_ value (1.9 μM) for Pi transport (Preuss et al., 2011). The expression locations and *K*_m_ value for Pi transport indicate the possible role of HvPHT1.1 in P uptake. *HvPHT1.6* is expressed in both roots and shoots (Huang et al., 2008). Also, it is highly expressed in old leaves compared to young leaves, especially in the leaf phloem tissue (Rae et al., 2003). HvPHT1.6 shows the linear transport activity for Pi-stimulated inward current over a concentration range of 5 to 30 mM in *Xenopus laevis* oocytes (Preuss et al., 2010). These results suggest that *HvPHT1.6* function as a low-affinity Pi transporter responsible for Pi remobilization in the whole plant. Huang et al. (2011) investigated the expression of *PHT1* genes and its relationship with P acquisition efficiency and P utilization efficiency (the amount of biomass produced per unit of acquired P) in four barley genotypes. They did not find a clear pattern in the expression of the four *HvPHT1.1* paralogs (*HvPHT1.1, 1.2, 1.9, 1.10*) among the four barley genotypes, but observed that the expression of *HvPHT1.3* and *1.6* positively correlated with P utilization efficiency. *HvPHT1.8* and *HvPHT1.11* (known as *HvPT11*) have been demonstrated to be specifically activated by arbuscular mycorrhizal (AM) fungi (Glassop et al., 2005; Sisaphaithong et al., 2012), indicating their possible roles in the mycorrhizal pathway of Pi uptake.

Wheat is one of the most important crops. However, limited attempts have been made to dissect the role of Pi transporters in wheat (Secco et al., 2017). Davies et al. (2002) isolated the first full-length sequence of a wheat *PHT1* gene (*TaPHT1.10-U*, formerly known *TaPT2*) and partial clones of several other putative *PHT1* genes. *TaPHT1.10-U* was induced by P-deficiency in roots, and had higher transcript abundance in P-efficient wheat varieties than in inefficient ones (Davies et al., 2002). In yeast, *TaPHT1.10-U* can complement high-affinity phosphate transporter gene *PHO84* function (Zeng et al., 2002) and shows an apparent mean *K*_m_ of 23.6 μM Pi (Guo et al., 2014). Overexpression of *TaPHT1.10-U* increases plant dry weight and Pi acquisition, whereas knock-down of *TaPHT1.10-U* has the opposite effect (Guo et al., 2014). These results suggest that *TaPHT1.10-U* functions as a high-affinity Pi transporter and mediates Pi uptake. A recent study observed that *TaPHT1.12-7A* (former name *TaPHT1.4*) was root-specific and P-deficiency inducible. Yeast complement analysis showed that *TaPHT1.12-7A* encodes a high-affinity Pi transporter with an apparent *K*_m_ of 35.3 μM. Overexpressing *TaPHT1.12-7A* significantly improves growth traits and accumulates more Pi than the wild-type plant and those with downregulated *TaPHT1.12-7A* expression (Liu et al., 2013). A recent study also revealed the relationships between *PHT1* expression and P use efficiency in wheat (Aziz et al., 2014). The highly P-efficient wheat cultivar Chinese 80-55 has a higher Pi acquisition in the presence of Pi and accumulates higher Pi concentrations in all organs upon Pi withdrawal compared with the less-efficient cultivar Machete. These differences correlate with differential organ-specific expression of Pi transporters *TaPHT1.10-4A* (reported as *TaPHT1.2*, GenBank: AY293828), *TaPHT1.6-5A* (reported as *TaPHT1.5*, GenBank: AF110180) and *TaPHT1.4-5B* (reported as *TaPHT1.8*, GenBank: AK333026) (Aziz et al., 2014). Shukla et al. (2016) found that aleurone accumulates more Pi with higher expression of *TaPHT1* genes compared to endosperm. *TaPHT1.8-6A* (known as *TRIae; Pht1; myc*, Glassop et al., 2005, GenBank: AJ830009), *TaPHT1.11-4A* (known as *TRIae; Pht1; 12*, GenBank: AB753271), *TaPHT1.11-4B* (known as *TRIae; Pht1;11*, GenBank: AB753270), and *TaPHT1.11-4D* (known as *TRIae; Pht1; 10*, GenBank: AB753269) have been found to be induced by AM fungi (Glassop et al., 2005; Sisaphaithong et al., 2012). Although expression of some *PHT1* genes has displayed correlation with P use-related traits in wheat and its close relative barley under controlled conditions, an on-farm field-scale investigation is required to explore the *PHT1* genes contributing to P uptake and utilization, as the response of *PHT1* genes to P supply level under controlled conditions greatly differed from that under field conditions. Our recent study showed that the expression of *TaPHT1.1, 1.2, 1.9*, and *1.10* in roots at the flowering stage under low P conditions was lower than that under high P conditions in a field experiment (Teng et al., 2013). The inhibition of these four wheat genes by P-deficiency could be, at least partially, explained by the upregulated AM colonization under P-deficiency, considering that AM colonization has been found to inhibit the response of *HvPHT1.1* and *HvPHT1.2* to P deficiency in barley (Glassop et al., 2005).

In this study, we aimed to identify the sequences of *PHT1* genes in the whole genome of wheat, and to analyze the correlation between the *PHT1* expression and P uptake under field conditions. We isolated 21 full length sequences of *PHT1* genes in wheat, and further analyzed their functions, expression location and response to P supply level. We observed that the expression of *TaPHT1.1, 1.2, 1.9*, and *1.10* in roots at the flowering stage contributed to P uptake of different wheat varieties under field conditions.

## Materials and Methods

### Wheat Varieties

The winter wheat (*Triticum aestivum*) variety Xiaoyan 54 was commercially released in 2000, and was used to isolate *TaPHT1* sequences, and to analyze gene expression location and response to P availability. The winter wheat varieties Kenong 9204 (KN9204) and Shijiazhuang 8 (SJZ8) were commercially released in 2003, and were used in the field experiments to analyze the relationship between *TaPHT1* expression and P uptake.

### Isolation of PHT1 Pi Transporters in Wheat

To isolate *PHT1* sequences from the wheat variety Xiaoyan 54, we performed BAC library screening and genomic sequence amplification by using the primers in Supplementary Table S1. After several rounds screening the BAC library of Xiaoyan 54 (Dong et al., 2010), we obtained 28 BAC clones which contained *PHT1* genes. These BAC clones were sequenced commercially by using a Roche/454 GS-FLX Titanium System (Roche Diagnostics, Germany) at SinoGenoMax Co., Ltd. (Chinese National Human Genome Center, Beijing, China). The resultant sequences were examined for the promoter and protein-coding sequences of *PHT1* genes, and consequently the primers were designed to isolate the coding regions of the *PHT1* genes in these BAC clones. The PCR products amplified from BAC clones and genomic DNA were sub-cloned into a pMD18-T Vector (Takara Bio, Dalian, China), and then sequenced commercially at SinoGenoMax Co., Ltd. The putative *cis*-elements in the promoters were predicted by RSAT::Plants software^1^. We used the neighbor-joining method to generate a phylogenetic tree of PHT1 proteins from wheat, *Triticum urartu, Aegilops tauschii*, barley, maize (*Zea mays*), rice, and Arabidopsis, and the phylogenetic tree was drawn using MEGA 5.0 (Tamura et al., 2011). Sequence alignment was performed by DNAMAN6.0 (Lynnon BioSoft, San Ramon, CA, USA).

### Functional Complementation Assay of Pi Transporters in Yeast

The yeast manipulations were performed as previously described (Ai et al., 2009). For the complementation assay, the coding sequences of the *TaPHT1* genes were amplified by PCR and subcloned into the yeast expression vector p112A1NE to create *TaPHT1*-*p112A1NE* constructs. These constructs and the empty vector p112A1NE were transformed into the yeast Pi uptake-defective mutant MB192 (Bun-Ya et al., 1991). Because the *PHT1* transporters are members of the H^+^/Pi symporter family, we firstly evaluated the optimal pH value for the growth of the transformed and control yeast strains. After measuring the optical density of the yeast cell lines at pH values ranging from 4 to 8 in yeast nitrogen base (YNB) liquid medium, we observed that the optimal pH value for most of the yeast mutant cells carrying *TaPHT1*s was 6, whereas the optimal pH value for the wild-type ranged from 4 to 6. Therefore, the pH value was set to 6 in the subsequent studies. To measure the kinetic growth profiles of the yeast strains, the yeast cells were grown in YNB liquid medium to the logarithmic phase (when the absorbance at 600 nm was 0.8), and were then harvested and washed in Pi-free YNB medium. Then, the yeast cells were grown at 30°C for 24 h in the YNB liquid media containing 200 μM Pi (high Pi) and 20 μM Pi (low Pi). The absorbance at 600 nm (OD600) was recorded every 6 h. MB192 and p112A1NE were kindly provided by Prof. Shubin Sun from Nanjing Agricultural University, Nanjing, China.

### Plant Growth Conditions

A hydroponic culture and three field experiments were conducted. The winter wheat variety Xiaoyan 54 was used in the hydroponic culture. The nutrient solution and growth conditions of the hydroponic culture were described by Wang et al. (2013). The seedlings, after 6 days of germination, were grown in nutrient solutions that contained 200 μM Pi (high P) or 5 μM Pi (low P). The plants were grown at 20°C for 3 weeks, and the roots and shoots were collected separately for gene expression analysis.

The field experiment in the experimental station of the Institute of Genetics and Developmental Biology in Beijing was carried out in the 2012–2013 growing season. The plant density and P fertilizer treatments was described by Wang et al. (2013). Briefly, the low P and high P treatments, i.e., 0.0 g m^-2^ and 13.5 g m^-2^ of P as calcium superphosphate, respectively, were applied before sowing. The seeds of Xiaoyan 54 were sown at the end of September in 2012. At the re-greening stage (March 18, 2013), the roots in 0–30 cm depth soil and shoots were collected separately. At the flowering stage (May 3, 2013), the stems, spikes, flag leaves, and aging leaves (top third leaf) were sampled. At the grain filling stage (14 days after flowering), the stems, grains, flag leaves, and aging leaves were collected. In each sampling time, 10 plants were randomly selected in each of the three replications. The plant samples were stored at -80°C for gene expression analysis.

Two field experiments at the Quzhou Experiment Station (36.5° N 115.0° E, 40 m above sea level) of the China Agricultural University have been described by Teng et al. (2013). These two experiments were conducted in the 2009–2010 growing season (referred as the 2010 field experiment) and the 2010–2011 growing season (referred as the 2011 field experiment). The data were collected from the winter wheat varieties KN9204 and SJZ8 at the P application rates 0, 100, and 400 kg ha^-1^ of P as calcium superphosphate (referred as P0, P100, and P400, respectively). The data for P use-related traits and expression levels of *TaPHT1* genes in KN9204 have been reported by Teng et al. (2013). The P application rates P0, P100, and P400 represented deficient, optimal, and excessive P supply, respectively (Teng et al., 2013).

### RNA Extraction and Quantitative Real-time PCR

Total RNA extraction and real-time quantitative reverse transcription PCR (qRT-PCR) were performed according to the methods of Teng et al. (2013). The primer sequences are listed in Supplementary Table S2. The gene expression levels were normalized to the internal control of *TaActin*.

### Measurement of Total P Concentration in Plant Samples

To determine plant total P, dried samples were milled and subsequently digested with concentrated H_2_SO_4_ and H_2_O_2_ using the molybdate-blue colorimetric method (Murphy and Riley, 1962).

### Statistical Analysis

The SPSS statistical software (SAS Institute, Cary, NC, USA) was used to perform analysis of variance using one-way analysis of variance (ANOVA). Comparisons of means were performed using Duncan’s multiple range analysis test and paired samples *t*-test (α = 0.05).

## Results

### Sequence Analysis of PHT1 Transporters in Wheat

We cloned 21 *TaPHT1* genes from common wheat through screening a BAC library of Xiaoyan 54 and amplifying genomic sequences (Supplementary Table S3). None of these genes contained intron, 19 of them contained full length ORFs, and their deduced protein sequences varied from 521 to 539 amino acids (Supplementary Table S3). One nucleotide deletion occurred at 368 bp downstream of the start codon in *TaPHT1.10-4B* and thus resulted in a frame shift mutation, and *TaPHT1.9-4A* had a premature stop codon mutation at 810 bp downstream of the start codon, but this premature stop mutation was not found in the Chinese spring. We mapped the cloned *TaPHT1s* on chromosomes by sequence analysis of BAC contigs and the reference sequence of Chinese spring^2^. The five clones, BAC48, BAC470, BAC674, BAC1217, and BAC1779, formed a BAC contig which contained *TaPHT1.1-4B, 1.2-4B, 1.9-4B*, and *1.10-4B* (Supplementary Figure S1A). *TaPHT1.9-4B* and *1.2-4B* matched with the sequences from 210,450 to 212,013 bp and from 299,422 to 300,999 bp in the scaffold TGACv1_scaffold_320302_4BL, respectively (Supplementary Figure S1B), Therefore *TaPHT1.1-4B* and *1.10-4B* were assigned to chromosome 4B. Further sequence analysis showed that the 1011 bp fragment from 220136 to 221146 bp and the 1316 bp fragment from 258319 to 259634 bp of the scaffold TGACv1_scaffold_320302_4BL matched with *TaPHT1.10-4B* and *1.1-4B*, respectively, but both fragments had low sequence quality. The former fragment contained 585 unknown nucleotides, and the later fragment contained 1004 unknown nucleotides; this was possibly why these two fragments were not annotated. *TaPHT1.10-4B* also showed 99.7% of sequence identity with the sequence from 1 to 1465 bp in the scaffold TGACv1_scaffold_684896_U (Overlapping gene TRIAE_CS42_U_TGACv1_684896_AA2159320, Supplementary Table S3). *TaPHT1.10-4B* from Xiaoyan 54 seemed to be the allele of TRIAE_CS42_U_TGACv1_684896_AA2159320 from Chinese spring, as both genes had the nucleotide deletion at 368 bp downstream of the start codon.

Genome-wide analysis of the genome sequence in Triticum_aestivum_CS42_TGAC_v1 assembly for Chinese spring^2^ totally identified 32 Gene IDs for *TaPHT1* (Supplementary Table S3). The Gene ID TRIAE_CS42_4BL_TGACv1_320302_AA1034400 matched with *TaPHT1.2-4B* and *TaPHT1.9-4B*; and no Gene ID was found to match with *TaPHT1.1-4B* and *TaPHT1.10-U* cloned in the current study or *TaPHT1.11-4B* (former name *TRIae; Pht1;11*) cloned by Sisaphaithong et al. (2012). Therefore, we identified a total of 36 *TaPHT1* genes (Supplementary Table S3). The 31 genes of *TaPHT1.1*-*TaPHT1.11* were named according to their similarity with barley *PHT1* transporters and chromosome location, and the remaining five genes were sequentially named *TaPHT1.12, TaPHT1.13*, and *TaPHT1.14*, together with the chromosome location (Supplementary Table S3). The *TaPHT1* genes were unevenly distributed on the chromosomes, as there were 17 and 8 *PHT1* genes on the chromosomes of homologous group 4 and 5, respectively (Supplementary Table S3). This uneven distribution was mainly due to the *PHT1* clusters on the chromosomes of these homologous groups. For example, we found five *TaPHT1* genes (*TaPHT1.5-4B, TaPHT1.1-4B, 1.2-4B, 1.9-4B* and *1.10-4B*) within a 150-kb region on the long arm of chromosome 4B (Supplementary Figure S1). The scaffold TGACv1_scaffold_407907_5BL on the long arm of chromosome 5B conferred *TaPHT1.3-5B* and *TaPHT1.4-5B* within an approximate 18-kb region (Supplementary Table S3). We also cloned the promoter sequences of 10 *TaPHT1* genes, and all these promoters were found to contain several putative Pi-starvation response regulator PHR1 binding *cis*-element P1BS and WRKY transcription factor binding element W-Box (Supplementary Figure S2).

**FIGURE 1:**
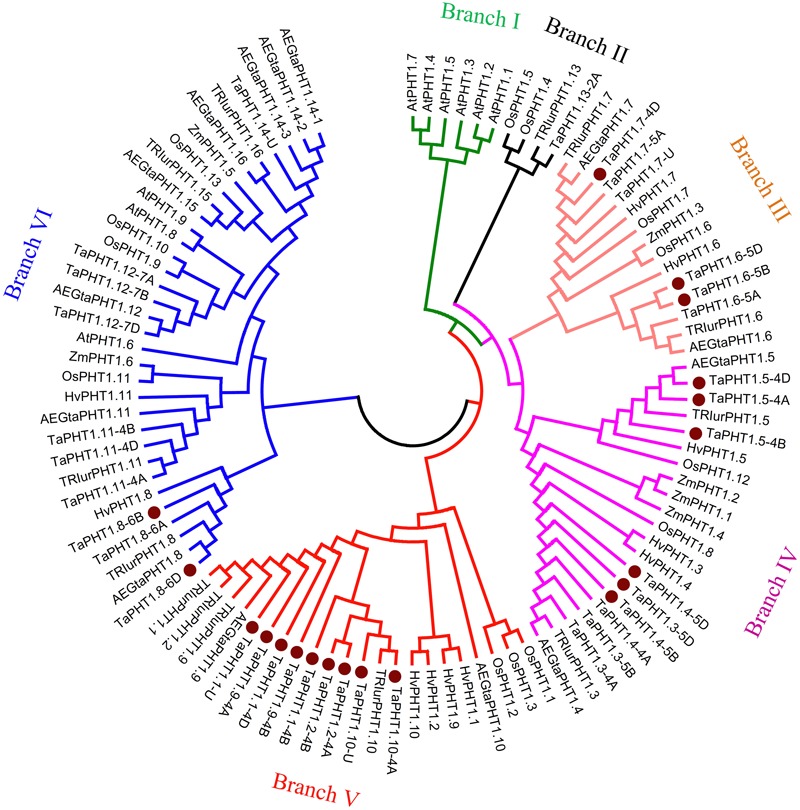
**Phylogenetic analysis for PHT1 transporters in plants.** The neighbor-joining method was used to perform a phylogenetic analysis of PHT1 transporters from *Triticum aestivum* (Ta), *Triticum urartu* (TRIur), *Aegilops tauschii* (AEGta), *Hordeum vulgare* (Hv), *Oryza sativa* (Os), *Zea mays* (Za), and *Arabidopsis thaliana* (At). Boot strap values are from 1000 replications. The genes cloned in the present study are indicated by a dot.

We calculated the relatedness of TaPHT1s using the ClustalX 2.1 software, with the results suggesting that the protein sequence identities ranged from 46 to 99%. The highest identities were found between the protein sequences of TaPHT1.1/1.2/1.9/1.10, and for that of TaPHT1.3/1.4. There were more than 98% of protein sequence identities between each other of the homologous alleles at a given *TaPHT1* locus from genomes A, B, and D (e.g., *TaPHT1.8-6A, -6B* and *-6D*). A neighbor-joining tree was constructed using a multiple sequence alignment according to TaPHT1 proteins and the PHT1 sequences from *Triticum urartu, Aegilops tauschii*, barley, maize, rice, and Arabidopsis (Supplementary Table S3). *TaPHT1.10-4B* was not included in the phylogenetic analysis, as it contained a frame shift mutation. The 35 TaPHT1s were clustered into five of the six branches (**Figure 1**). Branch I only contained PHT1s from Arabidopsis. TaPHT1.13-2A, TRIurPHT1.13, and OsPHT1.4/1.5 formed Branch II. The six TaPHT1.6/1.7 genes fell into Branch III which contained HvPHT1.6/1.7 and OsPHT1.6/1.7. The nine TaPHT1.3/1.4/1.5 genes belonged to Branch IV, and showed a close relationship with HvPHT1.3/1.4/1.5. The nine TaPHT1.1/1/2/1/9/1.10 genes belonged to Branch V, and they closely related to HvPHT1.1/1.2/1.9/1.10 and OsPHT1.1/1.2/1.3. The 10 TaPHT1.8/1.11/1.12/1.14 genes were grouped into Branch VI, which contained the AM fungi-inducible PHT1s from cereals as well as AtPHT1.6/1.8/1.9 from Arabidopsis.

### Analysis of Pi Transport Activities of TaPHT1s in a Yeast Strain Defective in Pi Uptake

**FIGURE 2:**
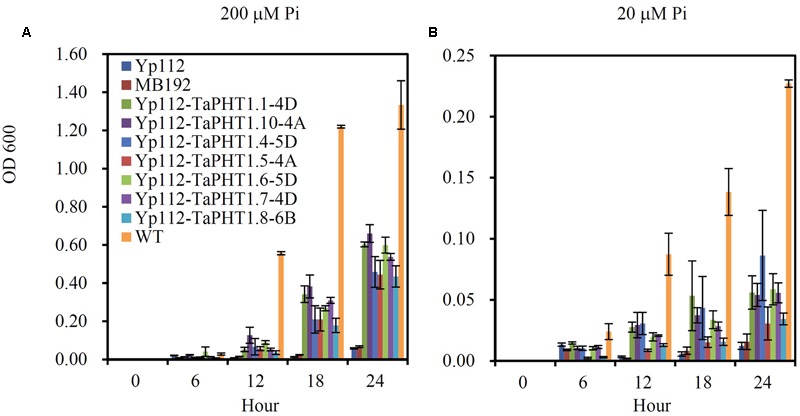
**Functional expression of seven *TaPHT1* genes in yeast.** Kinetic growth profiles of the wild-type (WT), MB192, MB192 transformed with an empty expression vector (Yp112) and the candidate *PHT1* genes (Yp112-*TaPHT1s*) generated from a 24 h YNB culture medium under 200 μM Pi **(A)** and 20 μM Pi **(B)**. OD 600, Optical density at 600 nm. Data are mean ± SE of three biological replications.

We analyzed the Pi transport activities of *TaPHT1.1-4D, 1.10-4A, 1.4-5D, 1.5-4A, 1.6-5D, 1.7-4D*, and *1.8-6B* genes using the yeast mutant MB192 strain (*pho84* mutant; Bun-Ya et al., 1991), which is defective in Pi uptake. *TaPHT1.1-4D* and *1.10-4A* were selected to represent the closely related *TaPHT1.1*/*1.2*/*1.9*/*1.10* which encoded two types of protein length, 521 amino acids and 525 amino acids. *TaPHT1.4-5D* was chosen to represent the closely related *TaPHT1.3*/*1.4*. The coding regions of the seven selected *TaPHT1* genes were separately inserted into the yeast expression vector p112A1NE under the control of the yeast alcohol dehydrogenase promoter. The constructs were separately transformed into a yeast Pi transporter mutant MB192. An empty vector was also transformed to be used as a control (Yp112). We first analyzed the complementation of MB192 by *TaPHT1* genes by using dilution based plate assays. All the yeast transformants harboring the candidate *TaPHT1* genes grew better than the Yp112 (empty vector control), but poorer than the wild-type in the plates which contained 20, 60, 100, and 140 μM Pi when the yeast cells were diluted to 1/100 OD value (Supplementary Figure S3C). This result indicated that the seven tested *TaPHT1* genes could partially restore the growth of MB192 mutant cells. Staining test for acid phosphatase activity also showed that *TaPHT1.6-5D* and *TaPHT1.10-4A* partially restore the growth of MB192 mutant (Supplementary Figures S3A,B). We then assessed the kinetic growth of the yeast cells in YNB liquid medium that contained 200 μM Pi (high Pi) and 20 μM Pi (low Pi). The wild-type yeast strain grew much quicker than the Yp112, MB192, and the yeast cells transformed with *TaPHT1* genes (Yp112-*TaPHT1*s), whereas Yp112 and MB192 exhibited a growth defect on both high Pi and low Pi media (**Figure 2**). All the yeast mutant cells carrying Yp112-*TaPHT1*s transformants grew faster than Yp112 and MB192 under high Pi and low Pi conditions (**Figure 2**), suggesting that these selected *TaPHT1*s had Pi transport activity.

### Responses of *TaPHT1* Expression to P Availability

**FIGURE 3:**
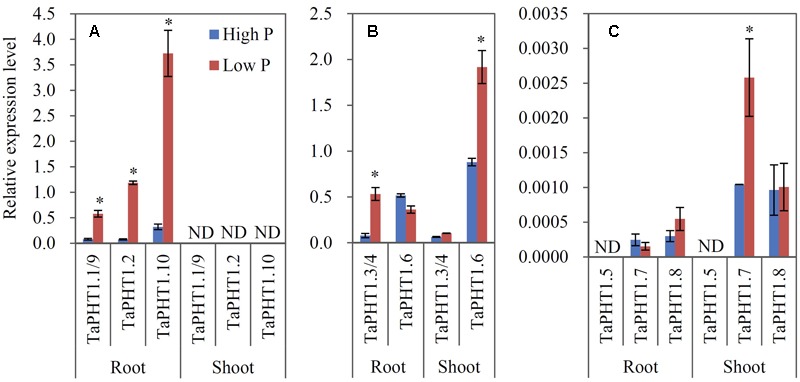
**Relative expression levels of *TaPHT1* genes in the roots and shoots of Xiaoyan 54 grown in high P and low P nutrient solutions in the hydroponic culture experiment at the seedling stage.** The seeds of Xiaoyan 54 were germinated at 25°C for 5 days, then transferred a nutrient solution containing 200 μM Pi (high P) or 5 μM Pi (low P). The plants were grown at 20°C for 3 weeks, and the roots and shoots were collected separately for gene expression analysis. The gene expression levels were normalized to the internal control of *TaActin*. **(A)** Relative expression levels of *TaPHT1.1/1.9, 1.2*, and *1.10*; **(B)** Relative expression levels of *TaPHT1.3/1.4*, and *1.6*; **(C)** Relative expression levels of *TaPHT1.5, 1.7*, and *1.8*. Data are mean ± SE of three biological replications. ^∗^ indicates significant differences between different P application rates (*P* < 0.05). ND, not detectable.

**FIGURE 4:**
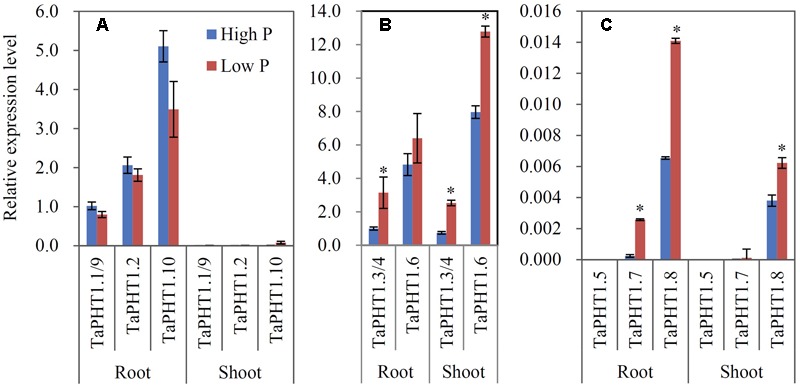
**Relative expression levels of *TaPHT1* genes in the roots and shoots of Xiaoyan 54 plants grown in the high P and low P soils in the field experiment in Beijing at the re-greening stage. (A)** Relative expression levels of *TaPHT1.1/1.9, 1.2*, and *1.10*; **(B)** Relative expression levels of *TaPHT1.3/1.4*, and *1.6*; **(C)** Relative expression levels of *TaPHT1.5, 1.7*, and *1.8*. The gene expression levels were normalized to the internal control of *TaActin*. Data are mean ± SE of three biological replications. ^∗^ indicates significant differences between different P application rates (*P* < 0.05).

Quantitative real-time RT-PCR was used to analyze the responses of *TaPHT1* genes to P supply levels at the seedling stage in a hydroponic culture and at the re-greening stage in a field experiment. Primers were designed to amplify the homologous alleles at a particular locus; for example, the relative expression level of *TaPHT1.2* might represent that of all three homologous alleles of *TaPHT1.2* (*TaPHT1.2*-*4A, -4B*, and *-4D*). In both the hydroponic culture and the field experiment, the expression of *TaIPS1.1*, a molecular indicator of plant Pi status (Teng et al., 2013), was upregulated by the low P treatment (Supplementary Figures S4A–C), indicating that the plants in the low P treatment in both of the experiments were P-starved. *TaPHT1.1/1.9, TaPHT1.2*, and *TaPHT1.10* were predominantly expressed in roots in both experiments (**Figures 3A, 4A**), and their expression was dramatically induced by low P treatment in the hydroponic culture (**Figure 3A**), but not in the field experiment (**Figure 4A**). Of these four root-specific genes, *TaPHT1.10* displayed the highest expression and *TaPHT1.1/1.9* the lowest (**Figures 3A, 4A**). *TaPHT1.3/1.4* and *TaPHT1.6* were expressed in both roots and shoots, and *TaPHT1.6* exhibited stronger expression than *TaPHT1.3/1.4* in both experiments (**Figures 3B, 4B**). These three genes differed in the response to P supply. Compared to high P treatment, low P treatment upregulated *TaPHT1.3/1.4* in roots and *TaPHT1.6* in shoots in the hydroponic culture (**Figure 3B**), and upregulated *TaPHT1.3/1.4* in roots and shoots and *TaPHT1.6* in shoots in the field experiment (**Figure 4B**). *TaPHT1.5, 1.7* and *1.8* were presented at very low expression levels in both roots and shoots in both of the experiments (**Figures 3C, 4C**). Upregulation by low P treatment was observed for *TaPHT1.7* in shoots in the hydroponic culture (**Figure 3C**), *TaPHT1.7* in roots and *TaPHT1.8* in roots and shoots in the field experiment (**Figure 4C**).

Since *TaPHT1.6* had the most abundant transcripts in shoots among the investigated *TaPHT1* genes, we further analyzed the expression of *TaPHT1.6* in different aerial parts at the flowering and grain filling stages (14 days after flowering) in the field experiment. The expression of *TaPHT1.6* was much higher in leaves than in stems, spikes, and grains, and was higher in aging leaves than in flag leaves (Supplementary Figure S5). Significant upregulation by low P treatment was observed in aging leaves, stems, spikes, and grains (Supplementary Figure S5).

### Relationship of *TaPHT1s* Expression with P Uptake

**FIGURE 5:**
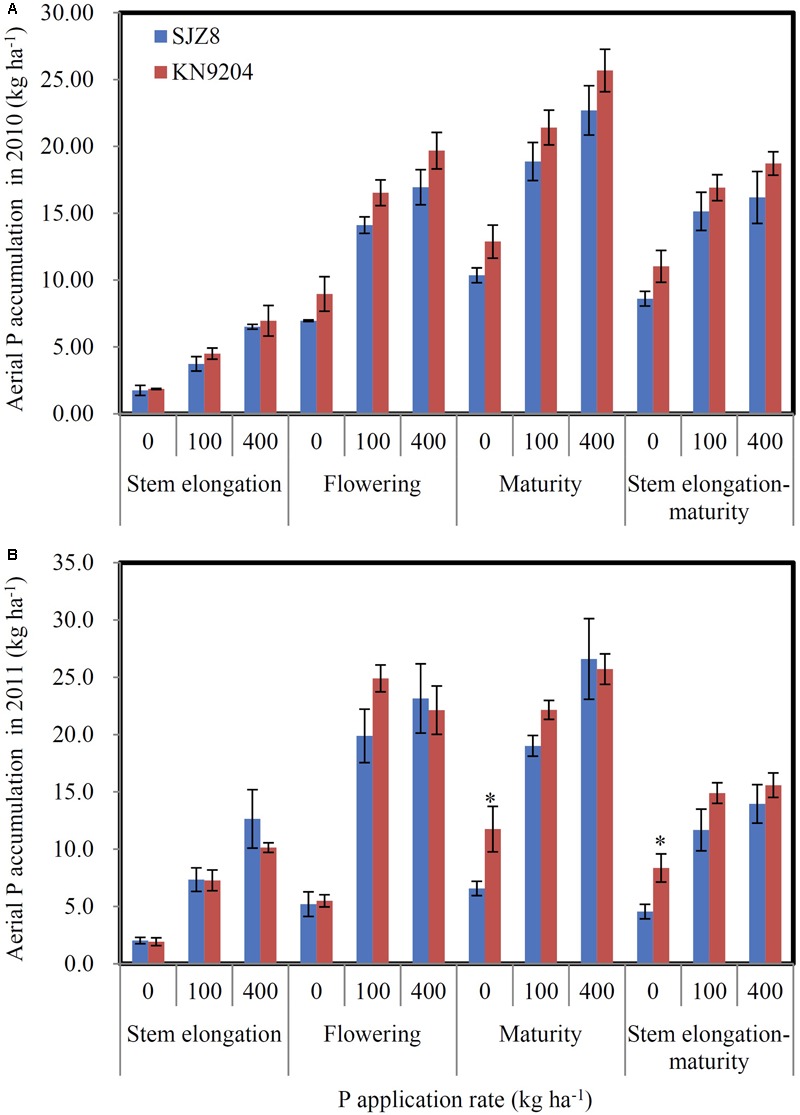
**P uptake of KN9204 and SJZ8 under different P application rates in the 2010 and 2011 field experiments in Quzhou. (A,B)** Aerial P accumulation in the 2010 **(A)** and 2011 **(B)** field experiments. 0, 100, and 400 indicate P application rate in kg P ha^-1^. Data are mean ± SE of four replicates. ^∗^ indicates significant differences between the two wheat varieties (*P* < 0.05).

We measured P uptake of two commercial wheat varieties at stem elongation, flowering, and maturity stages in two consecutive field experiments (2010 experiment and 2011 experiment). Data were collected at the P application rates of 0 kg P ha^-1^ (P0), 100 kg P ha^-1^ (P100), and 400 kg P ha^-1^ (P400). In most cases, the wheat variety KN9204 had higher total P concentration in shoots at stem elongation and flowering and in straws and grains at maturity than the wheat variety SJZ8, except for that of stem elongation in the 2011 experiment (**Table 1**). Comparison of aerial P accumulation between these two varieties showed that KN9204 absorbed more P than SJZ8 after stem elongation at all the P application rates in both of the field experiments (**Figure 5**).

**Table 1:** Total P concentrations in aerial parts in wheat plants in the 2010 and 2011 field experiments.

Sampling time	Tissue	P rate (kg ha^-1^)	P concentration (mg g^-1^) 2010		P concentration (mg g^-1^) 2011	
			SJZ8	KN9204	SJZ8	KN9204
Stem elongation	Shoot	0	2.13 ± 0.06^a^	1.91 ± 0.04^b^	1.51 ± 0.05	1.50 ± 0.12
		100	2.56 ± 0.09	2.74 ± 0.09	2.49 ± 0.31	2.18 ± 0.13
		400	3.46 ± 0.19	3.65 ± 0.09	3.17 ± 0.19	3.17 ± 0.21
Flowering	Shoot	0	2.13 ± 0.09	2.28 ± 0.16	1.32 ± 0.16	1.46 ± 0.08
		100	2.41 ± 0.03	2.42 ± 0.08	1.85 ± 0.07	2.04 ± 0.12
		400	2.51 ± 0.11	2.80 ± 0.09	2.18 ± 0.15	2.30 ± 0.14
Maturity	Grain	0	2.75 ± 0.06^b^	3.29 ± 0.05^a^	2.25 ± 0.07^b^	2.68 ± 0.14^a^
		100	3.17 ± 0.05^b^	3.56 ± 0.07^a^	2.79 ± 0.06^b^	3.38 ± 0.11^b^
		400	3.35 ± 0.08^b^	3.87 ± 0.10^a^	3.28 ± 0.10	3.36 ± 0.08
	Straw	0	0.29 ± 0.02	0.35 ± 0.04	0.20 ± 0.02	0.22 ± 0.01
		100	0.34 ± 0.03^b^	0.48 ± 0.04^a^	0.32 ± 0.04	0.39 ± 0.05
		400	0.42 ± 0.02^b^	0.55 ± 0.05^a^	0.44 ± 0.06	0.43 ± 0.04

*Different letters (a and b) indicate significant difference between SJZ8 and KN9204 at *p* < 0.05 level.*

**FIGURE 6:**
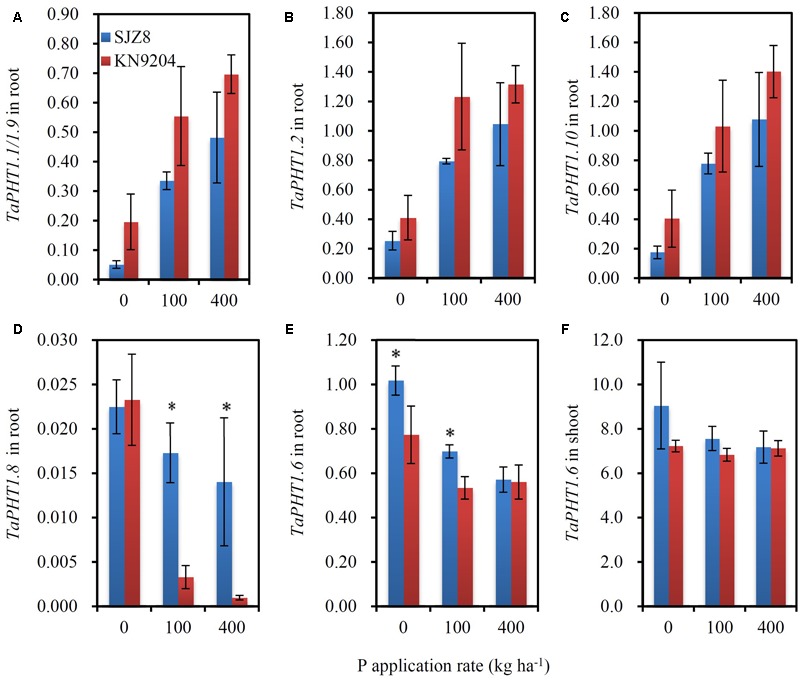
**The relative expression levels of *PHT1* genes in KN9204 and SJZ8 at the flowering stage under different P application rates in the 2011 field experiment in Quzhou. (A–D)** Relative expression levels of *TaPHT1.1/1.9* **(A)**, *TaPHT1.2* **(B)**, *TaPHT1.10* **(C)**, and *TaPHT1.8* **(D)** in roots; **(E,F)** Relative expression levels of *TaPHT1.6* in roots **(E)** and shoots **(F)**. The gene expression levels were normalized to the internal control of *TaActin*. Data are mean ± SE of four replicates. ^∗^ indicates significant differences between the two wheat varieties (*P* < 0.05).

**FIGURE 7:**
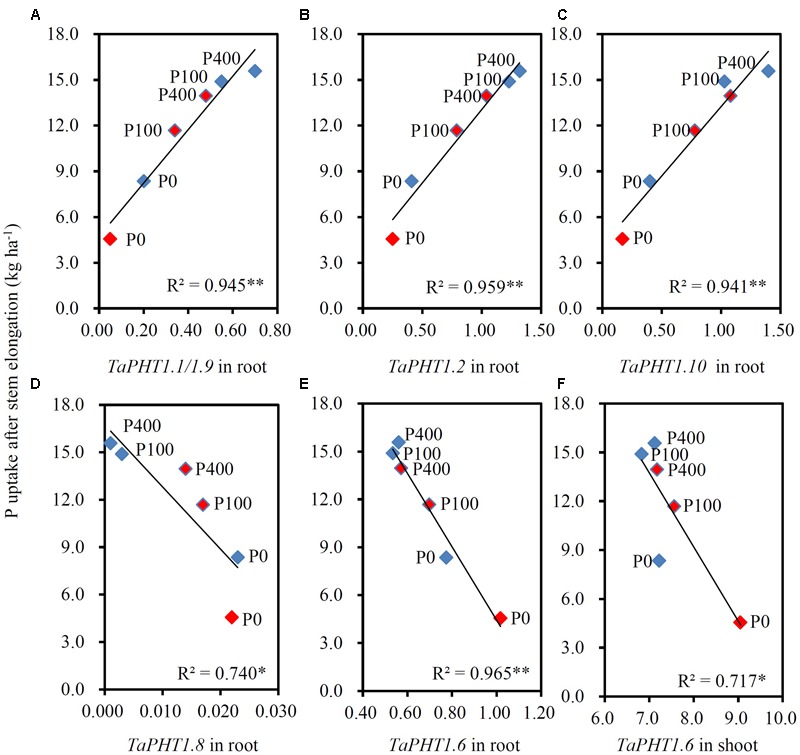
**Correlations between P uptake after stem elongation and the relative expression level of *TaPHT1* genes at flowering stage in the 2011 field experiment in Quzhou. (A–D)** Correlation of P uptake after stem elongation with relative expression levels of *TaPHT1.1/1.9* **(A)**, *TaPHT1.2* **(B)**, *TaPHT1.10* **(C)**, and *TaPHT1.8* **(D)** in roots; **(E,F)** Correlation of P uptake after stem elongation with relative expression levels of *TaPHT1.6* in roots **(E)** and shoots **(F)**. Blue and red diamonds indicate KN9204 and SJZ8, respectively. The data are the mean of four replicates. The data from KN9204 and SJZ8 are indicated by blue and pink color, respectively. P0, P100, and P400 indicate P application rates of 0, 100, and 400 kg P ha^-1^, respectively. ^∗^ and ^∗∗^ indicate the significance of *R*^2^ at *P* < 0.05 and *P* < 0.01, respectively.

As the differences in P uptake between KN9204 and SJZ8 were mainly observed at flowering and maturity, we analyzed the *TaPHT1*s expression at the flowering stage in the 2011 field experiment. The higher expression of *TaIPS1.1* at P0 than at P100 and P400 indicated that the wheat plants grown under P0 conditions were P-starved (Supplementary Figure S4D). *TaPHT1.1/1.9, 1.2*, and *1.10* were expressed more abundantly in the roots of KN9204 than in those of SJZ8 at all the three P rates (**Figures 6A–C**), whereas SJZ8 had higher expression of *TaPHT1.8* in roots at P100 and P400 (**Figure 6D**) and higher expression of *TaPHT1.6* in roots at P0 and P100 (**Figure 6E**) and in shoots at P0 than KN9204 (**Figure 6F**). The paired *t*-test showed that the mean values across the three P application rates for P uptake after stem elongation in 2010 and 2011 field experiments and the expression of *TaPHT1.1/1.9* and *1.10* at flowering in 2011 field experiment were significantly higher in KN9204 than in SJZ8 (Supplementary Table S4). We further analyzed the correlations between gene expression at flowering and P uptake after stem elongation (difference between stem elongation and maturity). P uptake after stem elongation showed a positive correlation with the expression of *TaPHT1.1/1.9* (**Figure 7A**), *TaPHT1.2* (**Figure 7B**), and *TaPHT1.10* in roots (**Figure 7C**), but a negative correlation with the expression of *TaPHT1.8* in roots (**Figure 6D**) and *TaPHT1.6* in roots and shoots (**Figures 6E,F**).

## Discussion

We identified a total of 36 *TaPHT1* genes named from *TaPHT1.1* to *TaPHT1.14* in wheat. Of the 32 *PHT1* genes with chromosome location information, 12, 11, and 9 were from the A, B, and D genomes (Supplementary Table S3), respectively. In order to evaluate the *PHT1* number in wheat, we also identified 13 *PHT1* genes in *Triticum urartu* and 14 *PHT1* genes in *Aegilops tauschii*, and these *PHT1* genes were named from *PHT1.1* to *PHT1.16* (Supplementary Table S3). We did not find the wheat Gene IDs which are orthologous to *PHT1.15* and *PHT1.16* of *Triticum urartu* and *Aegilops tauschii* (**Figure 1**), but several scaffolds from the short arms of group 2 chromosomes in wheat contained *PHT1.15* and *PHT1.6* like fragments which were not annotated yet. Although we identified a *PHT1.14* gene (*TaPHT1.14-U*) in wheat, there were three closely related *PHT1.14* genes (*AEGtaPHT1.14-1, 1.14-2*, and *1.14-3*) in *Aegilops tauschii* (**Figure 1**). Further detailed analysis of the Chinese spring genome sequence found that the forward orientation of seven fragments showed high similarity with *TaPHT1.14-U* in the scaffold TGACv1_scaffold_642582_U (Supplementary Figure S6). The second, fourth, fifth, and sixth fragments showed similarity only with the 3′-end of *TaPHT1.14-U* (Supplementary Figure S6). However, the first fragment showed 96–98% identity with 1–1604 bp of the 1656 bp coding region in *TaPHT1.14-U* (the third fragment 3, Supplementary Figure S6), whereas the seventh fragment located in the last 550 bp of the scaffold showed 99% identity with 1–550 bp of the coding region in *TaPHT1.14-U* (Supplementary Figure S6). As such, TGACv1_scaffold_642582_U may contain three closely related *PHT1.14* genes. Taking the information together, there may have as many as 16 (if with one *PHT1.14* gene)-18 (if with three *PHT1.14* genes) *PHT1* genes in each of the three subgenomes in wheat.

The cloned 10 *TaPHT1* promoters contained the putative Pi-starvation response regulator PHR1 binding *cis*-element P1BS and the WRKY transcription factor binding element W-Box (Supplementary Figure S2), indicating that *TaPHT1s* may be regulated by PHR1 and WRKY transcription regulatory factors. In fact, our previous study has documented that *TaPHR1* can form homodimers to activate *TaPHT1.10-U* expression *in vitro* (Wang et al., 2013). It has been reported that PHR1 regulates Pi starvation-inducible genes by binding as a dimer to the *cis*-element P1BS in the promoter region of its downstream gene (Rubio et al., 2001) and the majority of Pi starvation-inducible genes contain the P1BS element (Muller et al., 2007; Nilsson et al., 2010). As such, the P1BS elements in the promoters might contribute to the observed upregulation of *TaPHT1* genes by low P treatment (**Figure 3**). Several WRKY transcription factors have been found to bind to the W-box to regulate the expression of Pi-starvation response genes in Arabidopsis (Devaiah et al., 2007; Chen et al., 2009; Wang et al., 2014). Whether WRKY transcription factors involved in regulating the response of *TaPHT1* genes to P-deficiency is needed to be studied in the future.

The seven tested *TaPHT1* genes showed Pi-transport activity in yeast cells grown under low Pi and high Pi conditions (**Figure 2**). The genes from the same branch of the phylogenetic tree shared similar tissue-specific expression and response to P-deficiency (**Figures 3, 4**). The expression of *TaPHT1.1/1.2/1.9/1.10* in Branch V was root-specific and upregulated by low P treatment in the hydroponic culture (**Figure 3A**), but their upregulations by low P treatment was abolished in the field experiments (**Figures 4A, 6A–C**). These abolished upregulations by low P treatment were possible due to the increased AM colonization in roots under P deficiency (Teng et al., 2013), as AM colonization has been found to inhibit the response of *HvPHT1.1* and *HvPHT1.2* to P-deficiency in roots of barley (Glassop et al., 2005). In Branch V, *HvPHT1.1* has been found to encode high-affinity transporters of Pi (Preuss et al., 2011). Taking information together, TaPHT1 transporters in Branch V may function as high-affinity Pi transporters mediating Pi acquisition from soils. *TaPHT1.6* in Branch III and *TaPHT1.3/1.4* in Branch IV were expressed in both roots and shoots and were upregulated by low P treatment in the hydroponic culture and in the field experiment (**Figures 3B, 4B**). In aerial parts, *TaPHT1.6* was expressed in stems, leaves, spikes, and grains (Supplementary Figure S5). *HvPHT1.6* in Branch III was expressed in both roots and shoots (Rae et al., 2003). *OsPHT1.8* in Branch IV was expressed in various tissue organs from roots to seeds and plays an important role in Pi homeostasis and P redistribution from source to sink organs (Jia et al., 2011; Li et al., 2015). These results indicate that PHT1s in Branches III and IV may mediate Pi remobilization in whole plant. However, they may have diverse affinities for Pi, as OsPHT1.8 has been shown high-affinity for Pi (Jia et al., 2011), and HvPHT1.6 low-affinity for Pi (Rae et al., 2003). *TaPHT1.5* in Branch IV and *TaPHT1.7* in Branch III were expressed at very low levels in both of the hydroponic culture and field experiment (**Figures 3C, 4C**). The reported AM fungi inducible *PHT1*s were grouped into Branch VI (**Figure 1**), including *TaPHT1.8* and *TaPHT1.11* from wheat, *HvPHT1.8* and *HvPHT1.11* from barley, *ZmPHT1.6* from maize (Glassop et al., 2005; Sisaphaithong et al., 2012), and *OsPHT1.11* from rice (Paszkowski et al., 2002). Here, we found that *TaPHT1.8* was upregulated by low P treatment in the field experiments (**Figures 4C, 6D**), but not by low P treatment in the hydroponic culture (**Figure 3C**). Since we observed that low P treatment increased AM colonization rate in roots of KN9204 compared to high P treatment in field experiments (Teng et al., 2013), the upregulation of *TaPHT1.8* by low P treatment in the field experiments might reflect the fact that *TaPHT1.8* was exclusively induced by AM fungi (Glassop et al., 2005).

Previous studies state that transgenic modifying expression of *PHT1* genes altered P uptake and re-distribution in wheat (Liu et al., 2013) and rice (Ai et al., 2009; Jia et al., 2011; Yan et al., 2014). The transcript abundance of *PHT1* genes has been shown to relate with P uptake in wheat (Aziz et al., 2014) and P utilization efficiency in barley (Huang et al., 2011) under controlled conditions. These results indicate that mRNA levels of *PHT1* genes affect the capacities of P uptake and remobilization. Our current on-farm field-scale study showed that the expression of *TaPHT1.1/1.2/1.9/1.10* correlated with the differences in P uptake between different wheat varieties. The positive correlations between P uptake after stem elongation and the expression levels of *TaPHT1.1/1.2/1.9/1.10* at the flowering stage (**Figures 7A–C**) might result from two factors: P supply level and wheat variety. Firstly, both P uptake and expression of these *TaPHT1* genes increased with P application rate (**Figures 5, 6A–C**). Secondly, KN9204 had higher P uptake after stem elongation and the relative expression levels of *TaPHT1.1/1.2/1.9/1.10* at the flowering stage than SJZ8 at a given P application rate (**Figures 5, 6A–C**). In contrast to the positive correlations between the expression of *TaPHT1.1/1.2/1.9/1.10* and P uptake after stem elongation, the expression of *TaPHT1.8* and *TaPHT1.6* negatively correlated with P uptake after stem elongation (**Figures 7D–F**). The negative correlation between *TaPHT1.8* expression and P uptake after stem elongation resulted from the decreased *TaPHT1.8* expression with P application rate and lower *TaPHT1.8* expression in roots of KN9204 compared to that of SJZ8 at P100 and P400 (**Figure 7D**). However, this negative correlation did not support that AM colonization inhibited P uptake, as we did not analyze the expression of *TaPHT1.11* yet. It has been reported that *TaPHT1.11-A1, -B1*, and *-D1* were AM-inducible and were expressed at much higher level than *TaPHT1.8* (Sisaphaithong et al., 2012). The negative correlation between *TaPHT1.6* expression and P uptake after stem elongation mainly resulted from the decreased *TaPHT1.6* expression with P application rate (**Figures 7E,F**), as KN9204 and SJZ8 had similar expression levels of *TaPHT1.6* in roots at P400, and in shoots at P0, P100 and P400 (**Figures 6E,F**). Although *TaPHT1.6* may mediate P redistribution, the similar expression levels of *TaPHT1.6* in shoots at flowering did not explain the differences in grain P concentration between these two varieties (**Table 1**). This was possibly because that the transport of P to grains occurs during grain filling. As such, further research is needed to investigate the expression of *TaPHT1* genes including *TaPHT1.6* during grain filling, the research may identify the *TaPHT1* genes which contribute to the differences in grain P concentration between different wheat varieties.

In summary, the hexaploid wheat has many more *PHT1* genes than the diploid cereal crops such as barley and rice. Although we performed genome-wide analysis of *PHT1* genes, we did not isolate all the *PHT1* genes in wheat. The on-going wheat genome sequencing project will help us to understand the complexity of the Pi transport system in wheat. Although there were a large number of *PHT1* genes in wheat, the *TaPHT1* transporters from a given branch of the phylogenetic tree shared high similarities in sequences, expression locations, and responses to P-availability, this finding will help us to predict the roles of *TaPHT1* genes in mediating Pi uptake and re-distribution. Our research also provided useful cues to understand the influences of *PHT1* genes on the genotypic differences in P uptake. Further studies on mechanisms underlying the genotypic differences in *PHT1* expression will facilitate the breeding of wheat varieties with improve P use efficiency.

## Author Contributions

Y-PT, WT, and Y-YZ designed this study; Y-YZ screened BAC clones, WT and Y-YZ analyzed PHT1 sequences; Y-YZ and WT assayed expression and function of *PHT1* genes; all authors carried out the field experiments; WT and Y-YZ wrote the manuscript under the supervision of Y-PT. All authors have read and approved this manuscript.

## Conflict of Interest Statement

The authors declare that the research was conducted in the absence of any commercial or financial relationships that could be construed as a potential conflict of interest.

## Footnotes

1 http://floresta.eead.csic.es/rsat/

2 http://plants.ensembl.org/Triticum_aestivum/Info/Index
