# Supplementary material for: Genome-wide Identification, Characterization, and Expression Analysis of PHT1 Phosphate Transporters in Wheat
Source: Front Plant Sci. 2017 Apr 11;8:543. doi: 10.3389/fpls.2017.00543 (PMC5386973; doi:10.3389/fpls.2017.00543)
Supplement: Supplementary file 1 [file Presentation_1.pdf]

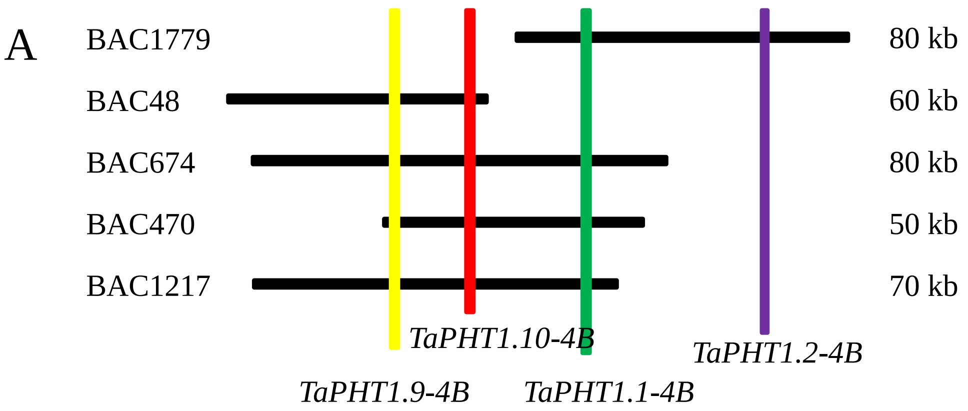

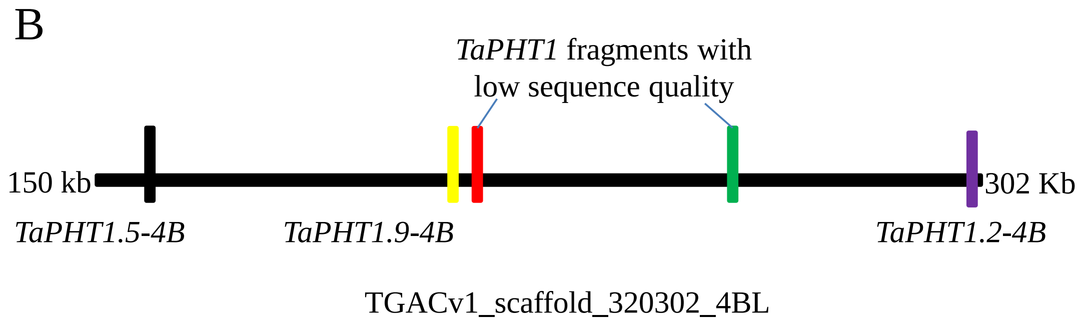

Figure S1. *PHT1* location sites in BAC clones from Xiaoyan 54 (A) and the scaffold TGACv1_scaffold_320302_4BL from Chinese spring (B).

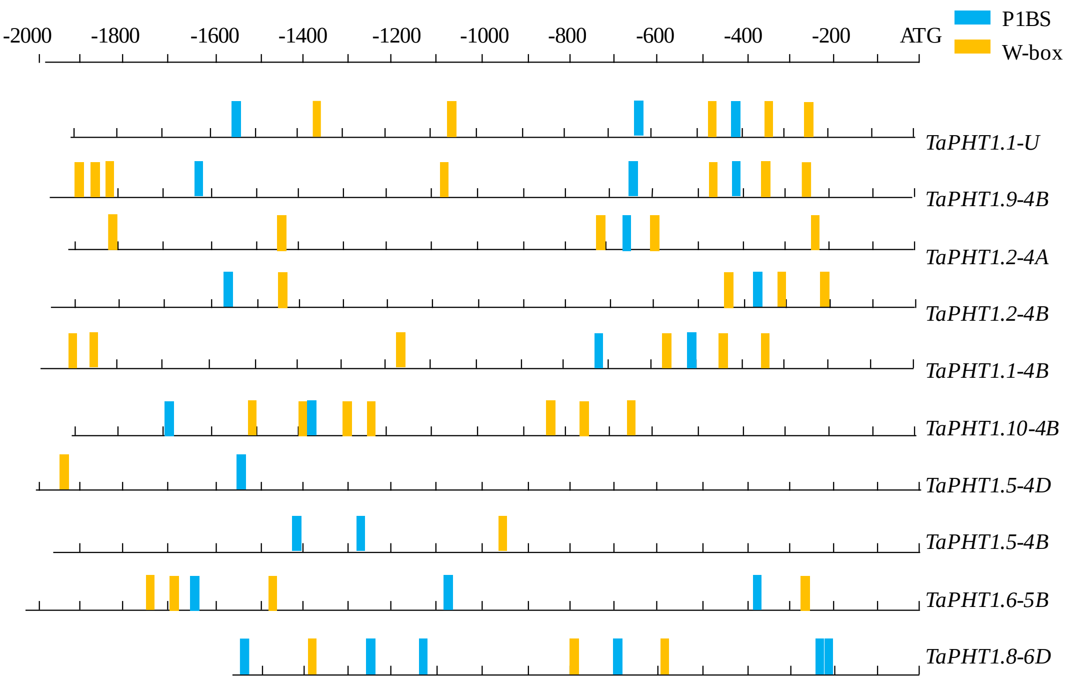

Figure S2. The cis-acting elements in the promoters of *TaPHT1* genes. P1BS, PHR1 binding element (GNATATNC); W-box, WRKY transcription factor binding element (TTGACY).

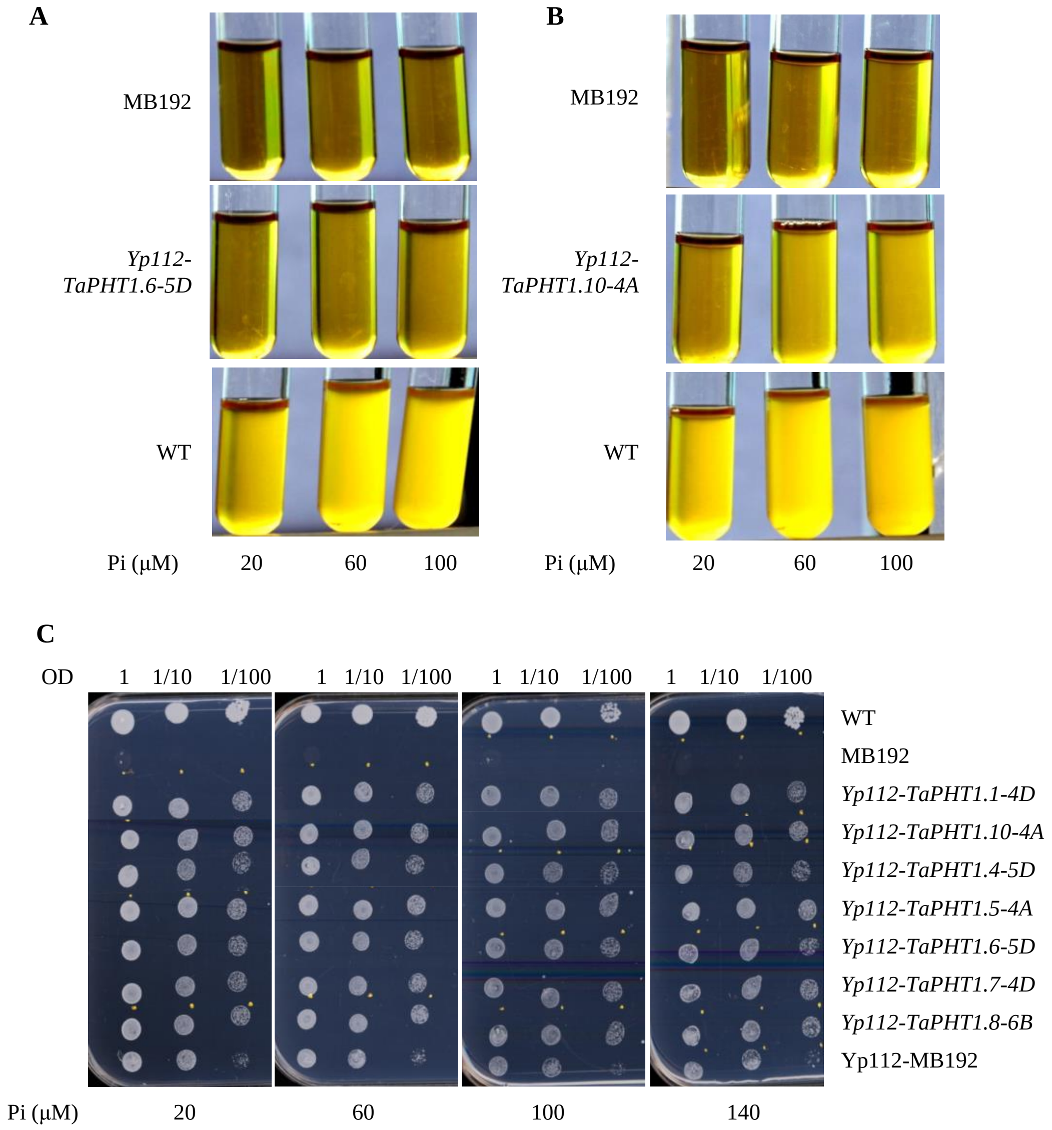

Figure S3. Functional analysis of seven *TaPHT1* genes in yeast. (A and B) Color shifts for acid phosphatase activity in the yeast strain MB192 (control), the wild type (WT), Yp112-*TaPHT1.6-5D* (A) and Yp112-*TaPHT1.10-4A* (B). The culture medium contains 20, 60, 100 μM Pi. **(C)** Growth profiles of the wild-type (WT), MB192, MB192 transformed with an empty expression vector (Yp112-MB192) and the candidate *PHT1* genes (Yp112-*TaPHT1s*) on synthetics defined (SD) Ura mediums under 20, 60, 100, and 140 μM Pi conditions. The plates were incubated at 30 °C for 3 d. Staining test for acid phosphatase activity was carried out according to the method described by Jia et al., 2011. Complementation of the pho84 mutant MB192 by *TaPHT1* genes was according to the method described by Qin et al., 2012.

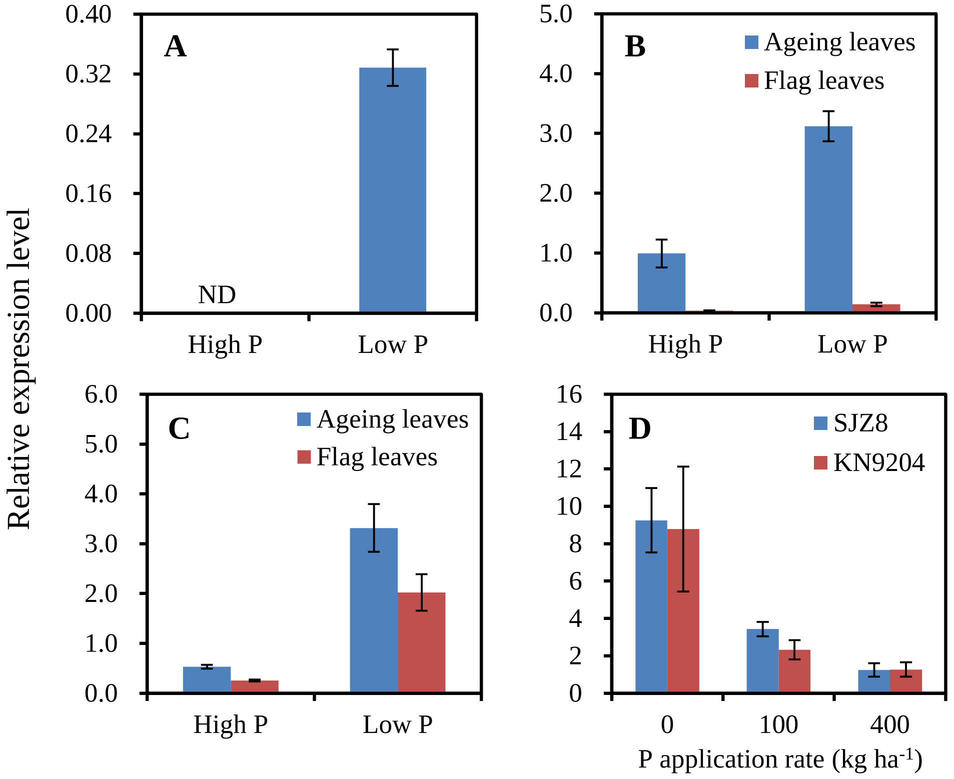

Figure S4. Relative expression levels of *TaIPS1.1* under different P supply levels. (A) Expression of *TaIPS1.1* in the shoots of Xiaoyan 54 grown in high P and low P nutrient solutions in the hydroponic culture experiment at the seedling stage. ND, not detectable. **(B and C)** Expression of *TaIPS1.1* in leaves of Xiaoyan 54 grown in the high P and low P soils in the field experiment in Beijing at flowering (B) and grain filling stage (C). **(D)** Expression of *TaIPS1.1* in the roots of KN9204 and SJZ8 at the flowering stage under different P application rates in the 2011 field experiment in Quzhou. The gene expression levels were normalized to the internal control of *TaActin*. Data are mean ± SE of three biological replications. Forward and reverse primers of *TaIPS1.1* were 5′-TCTCCTGTGAGTACCGGTGACA-3′, and 5′-ACTGTACACTAGTCGACAACTTGC-3′, respectively.

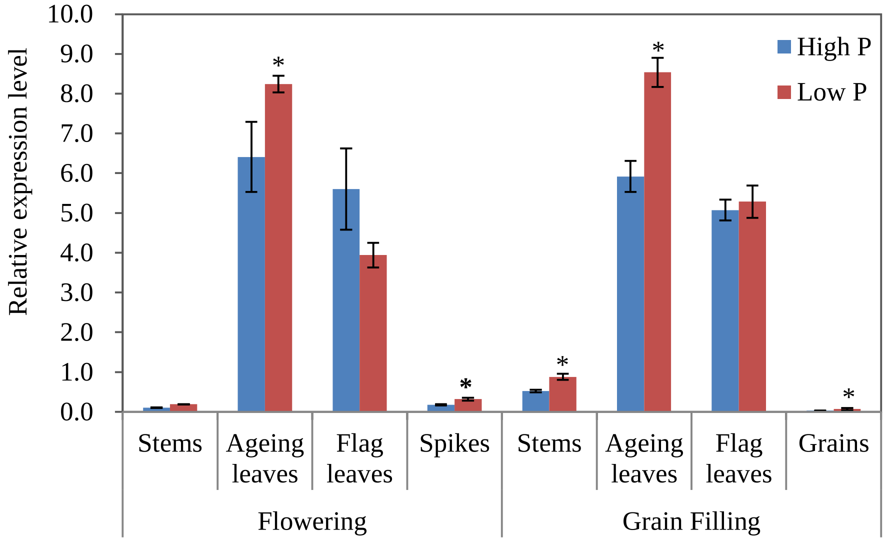

Figure S5. Relative expression levels of *TaPHT1.6* in aerial parts in Xiaoyan 54 grown in the high P and low P soils in the field experiment in Beijing at flowering and grain filling stage. The gene expression levels were normalized to the internal control of *TaActin*. Data are mean ± SE of three biological replications. * indicates significant differences between different P application rates ($P<0.05$).

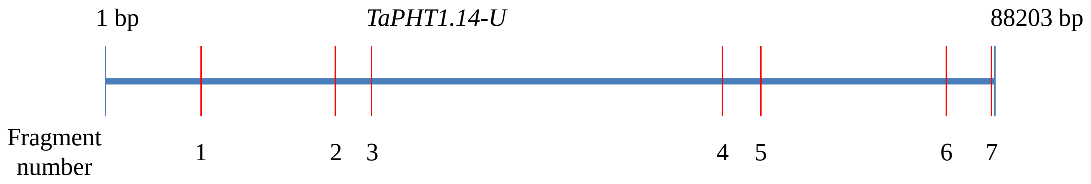

Fragment number	scaffold position (bp)	*TaPHT1.14-U* position (bp）	Identity
1	9482-10153	1-670	643/672(96%)
	10280-11216	668-1604	916/937(98%)
2	22959-23904	705-1650	822/948(87%)
3	26716-28516	1-1656	100%
4	61672-62247	1081-1656	546/576(95%)
5	65518-66421	705-1616	785/914(86%)
6	84146-85092	705-1650	820/949(86%)
7	87654-88203	1-550	546/550(99%)

Figure S6. Distribution of *TaPHT1.14-U* (1656 bp) like fragments in the Scaffold TGACv1_scaffold_642582_U (88203 bp). Red lines indicate the positions of the fragments highly similar with *TaPHT1.14-U*.

References

Jia, H., Ren, H., Gu, M., Zhao, J., Sun, S., Zhang, X., Chen, J., Wu, P., and Xu, G. (2011). The phosphate transporter gene *OsPht1;8* is involved in phosphate homeostasis in rice. *Plant Physiol* 156**,** 1164-1175.

Qin, L., Guo, Y.X., Chen, L.Y., Liang, R.K., Gu, M.A., Xu, G.H., Zhao, J., Walk, T., and Liao, H. (2012). Functional characterization of 14 *Pht1* family genes in yeast and their expressions in response to nutrient starvation in soybean. *PloS one* 7, e47726.
